# Production and Characterization of Calcium Silica Aerogel Powder as a Food Additive

**DOI:** 10.1021/acsomega.3c00358

**Published:** 2023-03-20

**Authors:** Burcu Karakuzu Ikizler, Emine Yapıcı, Sevil Yücel, Ertan Ermiş

**Affiliations:** † Bioengineering Department, Yildiz Technical University, Istanbul 34220, Turkey; ‡ Food Engineering Department, İstanbul Sabahattin Zaim University, Istanbul 34303, Turkey

## Abstract

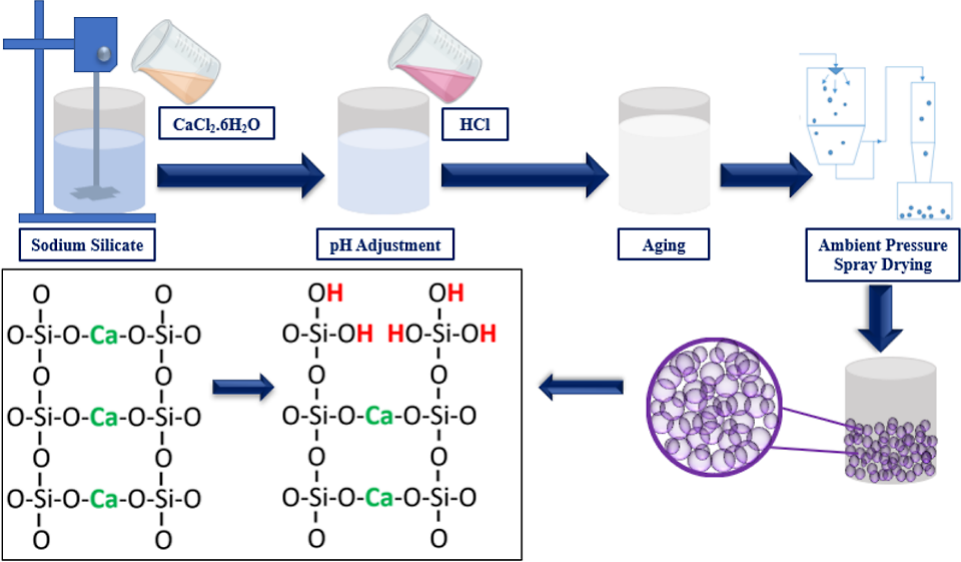

In this study, mesoporous calcium silica aerogels were produced for use as an anticaking food additive in powdered foods. A low-cost precursor (sodium silicate) was used, and calcium silica aerogels with superior properties were obtained with different pH values (pH 7.0 and pH 9.0) by modeling and optimizing the production process. The Si/Ca molar ratio, reaction time, and aging temperature were determined as independent variables, and their effects and interactions to maximize the surface area and water vapor adsorption capacity (WVAC) were evaluated by response surface methodology and analysis of variance. Responses were fitted with a quadratic regression model to find optimal production conditions. Model results showed that the maximum surface area and WVAC of the calcium silica aerogel that was produced with pH 7.0 were achieved at a Si/Ca molar ratio of 2.42, a reaction time of 5 min, and an aging temperature of 25 °C. The surface area and WVAC of calcium silica aerogel powder produced with these parameters were found to be 198 m^2^/g and 17.56%, respectively. According to the results of surface area and elemental analysis, calcium silica aerogel powder produced at pH 7.0 (CSA7) had the best results compared to that produced at pH 9.0 (CSA9). Therefore, detailed characterization methods were examined for this aerogel. The morphological review of the particles was performed with scanning electron microscopy. Elemental analysis was performed via inductively coupled plasma atomic emission spectroscopy. True density was measured in a helium pycnometer, and tapped density was calculated by the tapped method. Porosity was calculated using an equation using these two density values. The rock salt was powdered with a grinder and used as a model food for this study, and CSA7 was added at a rate of 1% by weight. The results showed that adding CSA7 powder to the rock salt powder at a rate of 1% (w/w) improved the flow behavior from the cohesive region to the easy-flow region. Consequently, calcium silica aerogel powder with a high surface area and high WVAC might be considered as an anticaking agent to use in powdered foods.

## Introduction

Calcium silicates are synthetically produced from silicon dioxide and calcium oxide with various ratios and are also obtained from naturally occurring limestone and diatomaceous earth.^1^ Calcium silicates, water-insoluble, white-colored, fine powder, are widely used in the industry. They can be used as a low-cost adsorbent to remove dissolved heavy metals in aqueous solutions.^1,2^ It is an important example of its widespread use as a safe alternative to asbestos for high-temperature insulation and fire resistance. In addition, calcium silicate materials can be used as absorbing, opacifying, and bulking agents in cosmetic product formulations (e.g., face powder) and as carriers in drug delivery systems due to their biocompatibility.^3^

According to the literature studies carried out so far, the silica aerogel has excellent properties such as low density, high specific surface area, high porosity, and low thermal conductivity, making it suitable for thermal insulation, sound insulation, catalysis, absorbent, and so forth. It finds a chance to be applied in different fields. In addition, it is possible to obtain silica aerogels doped with different metal/metal oxides (TiO_2_, Au, Mg, and Al), thanks to the functional groups of the silica aerogel that allow modification.

Calcium silicate contains 50–95% silicon dioxide (SiO_2_) and 3–35% calcium oxide (CaO) on the ignition base as per the Food and Agriculture Organization assay. The reasonable amount of usage in powdered foods does not exceed 2% by weight and does not exceed 5% by weight in baking powder.^1,4^

Agglomeration of food powders is an important problem that can occur during processing, transportation, and storage. With agglomeration, undesirable results such as difficulties in processing steps, deterioration of product quality, decrease in functionality, and shortening of shelf life may occur.^5^ Anticaking agents are added to food powders to prevent clumping, caking, or agglomeration and to improve their flow condition. In powdered foods consisting of crystalline structures, anticaking agents compete with the food powder for moisture and form protective physical barriers against moisture on the surface of hygroscopic particles. Another important approach in the working principle of anticaking agents is covering the food powder particles, eliminating friction between them, and stopping solid bridges’ formation by preventing crystal growth.^6−9^

Anticaking agents are crucial food additives for protecting powdery or granular food from any packaging problems to provide flowability. Moisture prevention is the main issue during the packaging, storage, and transportation of food granules. Also, usage of these agents in food powders that have mostly a hygroscopic structure inhibits microbial growth in food; thus, it is possible to extend the shelf life.^1^ In recent years, there has been an increasing interest in natural food additives. Health concerns (food allergies, etc.), dietary preferences (vegan nutrition, etc.), and religious restrictions of consumers affect food additive preferences. For example, many companies prefer nonanimal sources to produce stearate products. Silica aerogel products can be given as another example of novel anticaking agents. Silica aerogels have superior features and provide many advantages for use as an anticaking agent in food powders. High surface area, low bulk density, and very fine particle size are among their prominent features. Thus, even small amounts of silica aerogel powders can be used as an anticaking agent in food powders effectively.^10−12^

This study aimed to produce calcium silica aerogel powder which can be used as an alternative and innovative food additive, without using solvent exchange steps and expensive drying equipment. The parameters for calcium silica aerogel production (reaction time, molar ratio of silica and calcium, and aging temperature) were optimized for improving the properties of the aerogels via using the response surface methodology (RSM)—Box–Behnken approach.

## Experimental Procedure

Calcium chloride (CaCl_2_·6H_2_O) and hydrochloric acid (HCl, 65%) were purchased from Merck, Darmstadt, Germany. Sodium silicate solution, also called water glass (Na_2_O/3.2SiO_2_), was purchased from Palkim Chemistry, Turkey.

### Production of Calcium Silica Aerogels

Various methods and drying techniques have been reported in the literature to produce silica aerogels. In this study, production and drying methods were different from conventional procedures. The wet slurry that contains colloidal structures was formed by mixing calcium chloride salts with diluted sodium silicate solution to produce silica aerogel powders. The process continued with the implemented aging step to strengthen the molecular structure. The structure containing the calcium silica colloids was aged for 24 h. Then, it was washed with pure water and filtered to remove the excess salt in the aged structure. The process was continued until the salt was removed. After the washing process, the structure was dried in a spray dryer to perform the rapid evaporation process as an alternative to conventional aerogel drying processes such as supercritical drying or ambient-pressure drying following the solvent exchange method. Rapid evaporation gave a chance to protect the pores in the wet colloidal structures and prevent the collapse of the formed structure. The structure diluted with water sprayed from the spray dryer was quickly dried by making contact with the high-temperature air around the nozzle and collected in the sample storage chamber with the reverse airflow occurring inside the device. The suitable temperature for the drying process was selected as 190 °C. The outlet temperature was kept constant at 100° with the control of the airflow. The selected parameters that were thought to be effective during the production of the aerogels were the silicium calcium molar ratio (Si/Ca molar ratio of 1, 2, and 3), reaction time (5, 25, and 45 min), and aging temperature (25, 50, and 75 °C). The RSM along with the Box–Behnken method was employed by using Design-Expert software. In addition to these variables, the pH values of the prepared wet colloidal structures were adjusted to pH 7.0 for each batch produced with hydrochloric acid, and also for one set, they were produced without adding any acid solution (pH value around 9).

### Box–Behnken Experimental Design

The experimental design of calcium silica aerogels was prepared with three levels of the molar ratio of Si/Ca, aging temperature, and reaction time as given in Table 1. The three-level, three-factorial Box–Behnken experimental design with three replicates at the center point was used to evaluate the effect of the factors given in Table 1 on the results of the specific surface area and water vapor adsorption capacity (WVAC) of calcium silica aerogels. The molar ratio of Si/Ca (A), aging temperature (B), and reaction time (C) were designed for the statistical calculations of the three factors that were coded in three levels as −1 (low), 0 (central point), and +1 (high). The results were statistically evaluated to determine the effect of relations between the design factors per response by the analysis of variance (ANOVA) with the asset of Design-Expert software at the significance level when the *p*-value was greater than the 95% confidence limit.^13^

**Table 1:** Experimental Design of Optimization Studies with Variables, Factors, Levels, and Values

		levels		
code	factors	–1	0	1
A	Si/Ca molar ratio	1	2	3
B	aging temperature (°C)	25	50	75
C	reaction time (min)	5	25	45

## Characterization of Calcium Silica Aerogel Powder

The properties of the calcium silica aerogels were determined by several devices and techniques. The Brunauer–Emmett–Teller (BET) technique was used for determining the specific surface area. The Barrett–Joyner–Halenda (BJH) technique was used for pore size and pore volume analysis. The water vapor adsorption capacities of the synthesized calcium silica aerogels were investigated in a 70% relative humidity and room-temperature environment in a desiccator.^14,15^ Before starting the experiment, all samples were dried in an oven. The dry weight (*W*_d_) of the samples and the weight (*W*_s_) after 24 h of exposure to moisture were recorded. The water vapor adsorption capacities were calculated using eq 1.
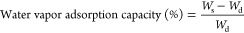
1

Aerogels were characterized by Fourier transform infrared (FTIR) spectroscopy coupled with an attenuated total reflection (ATR) unit. For IR measurements, absorption spectra (650–4000 cm^–1^) were recorded by SHIMADZU IR Prestige 21 (Japan). Calcium, silica, and sodium composition of calcium silica aerogels were determined by an inductively coupled plasma-optical emission spectrometer with good accuracy by PerkinElmer Optical Emission Spectrometer Optima 2100 DV (USA). The morphological properties of aerogel powders were observed by scanning electron microscopy (SEM). Images were recorded on Zeiss EVO LS 10 (Germany) with Au-coated samples. A Horiba LA 350 laser diffraction particle size distribution analyzer was used for the particle size distribution analysis. True (skeleton) density (*q*_s_) of silica aerogels was measured with a helium pycnometer (Pycnomatic ATC, Thermo Fisher Scientific, USA). The tapped density (*q*) of the silica aerogels was calculated with the compression of the known amount of powder in a measuring cylinder with 200 taps. After the tapping step, the mass was divided into the volume of powder and the results were obtained. The porosity values were calculated via the equation below (eq 2).
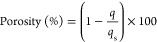
2

### Powder Flow Behavior Test

A powder flow tester (PFT) (Brookfield Engineering, UK) was used to determine the effect of the aerogel on the flowability of the selected model food powder. For this purpose, rock salt was ground in a centrifuge mill (Retsch ZM 200, Germany) and sieved (Retsch AS 200, Germany) to obtain the size fraction of 200–425 μm. A certain amount of calcium silica aerogel powder was added to rock salt powder to determine its effect on flowability.

The PFT device consists of a stationary lower sample cell and an upper lid having 18 blades to apply major principal stress (σ_1_) vertically at varying rates with downward linear movement, while at the same time, it causes shear stress in the powder sample with horizontal rotational movement. The basic measuring principle is based on measuring the unconfined yield stress (σ_c_) required for the powder material to begin to flow or deform after varying compression stress is applied. Five different consolidating stresses were applied to the powder bed in the measuring cell and a plot of σ_c_ against σ_1_ for each stress level generated a flow function curve. The linearized gradient is named as the flow function coefficient (ffc). ffc is equal to σ_1_/fc, the ratio of the major consolidation stress to the cohesive strength. A high ratio of ffc (>10) indicates a weak material (free flow), and a low value (<1) indicates a strong material (very cohesive).^16,17^

## Results and Discussion

### ANOVA and Optimization

The RSM with Box–Behnken design was used to understand the effect of selected production parameters such as the Si/Ca molar ratio, reaction time, aging temperature on the surface area, and WVAC of calcium silica aerogels that were produced with different pH values (pH 7.0 and pH 9.0). The Box–Behnken design matrix, independent variables, and actual and predicted values of dependent variables are demonstrated in Table 2. The actual values expressing the experimental test results and the predicted values expressing the statistical results are compared to each other. As a result, it can be stated that the actual and predicted values are remarkably close to each other.

**Table 2:** Box–Behnken Design Matrix and the Comparison of Observed and Predicted Results for Surface Area and Water Absorption Capacity of Calcium Silica Aerogels^a^

				dependent variables							
				surface area (m^2^ g^–1^)				WVAC (%)			
	independent variables			CSA7		CSA9		CSA7		CSA9	
no.	Si/Ca molar ratio	reaction time (min)	aging temperature (°C)	Act.	Pred.	Act.	Pred.	Act.	Pred.	Act.	Pred.
1	1.00	5.00	50.00	110.00	103.88	67.00	66.63	19.26	19.03	9.78	10.46
2	3.00	5.00	50.00	156.00	166.63	122.00	111.63	11.09	13.25	9.94	10.40
3	1.00	45.00	50.00	109.00	98.38	65.00	75.38	16.96	14.80	19.25	18.80
4	3.00	45.00	50.00	127.00	133.12	65.00	65.37	13.82	14.05	9.29	8.62
5	1.00	25.00	25.00	146.00	145.75	56.00	53.38	23.83	25.23	18.74	18.54
6	3.00	25.00	25.00	283.00	266.00	50.00	57.38	15.71	14.72	13.45	13.47
7	1.00	25.00	75.00	100.00	117.00	38.00	30.63	9.38	10.37	18.25	18.24
8	3.00	25.00	75.00	94.00	94.25	59.00	61.63	15.73	14.33	12.86	13.07
9	2.00	5.00	25.00	247.00	253.38	80.00	83.00	25.83	24.66	8.11	7.64
10	2.00	45.00	25.00	185.00	195.87	81.00	73.25	22.41	23.17	14.23	14.89
11	2.00	5.00	75.00	126.00	115.13	75.00	82.75	18.03	17.27	11.92	11.26
12	2.00	45.00	75.00	140.00	133.63	58.00	55.00	14.15	15.32	10.10	10.57
13	2.00	25.00	50.00	136.00	139.33	60.00	60.00	11.63	10.90	9.99	10.48
14	2.00	25.00	50.00	146.00	139.33	57.00	60.00	10.82	10.90	13.23	10.48
15	2.00	25.00	50.00	136.00	139.33	63.00	60.00	10.24	10.90	8.21	10.48

a Act.: actual, Pred.: predicted.

ANOVA was used for the accuracy and quality of the achieved models for each response, which are presented in Tables 3, 4, and 5. ANOVA was conducted at a significance level of α = 0.05 (confidence level of 95%). Also, in Table 3, the final equations in terms of coded factors and model summary statistics are presented.

**Table 3:** Final Equation in Terms of Coded Factors and Model Summary Statistics^a^

response	final equations in terms of coded factors and model summary statistics
CSA7 surface area	+139.33 + 24.37 × A – 9.75 × B – 50.13 × C – 7.00 × A × B – 35.75 × A × C + 19.00 × B × C – 16.29 × A^2^ + 2.46 × B^2^ + 32.71 × C^2^
	*R*^2^ 0.9675, adjusted *R*^2^ 0.9091, adequate precision 13.233
CSA7 WVAC (%)	+10.90 – 1.64 × A – 0.86 × B – 3.81 × C + 1.26 × A × B + 3.62 × A × C – 0.12 × B × C + 0.22 × A^2^ + 4.16 × B^2^ + 5.04 × C^2^
	*R*^2^ 0.9455, adjusted *R*^2^ 0.8475, adequate precision 9.064
CSA9 surface area	+60.00 + 8.75 × A – 9.38 × B – 4.63 × C – 13.75 × A × B + 6.75 × A × C – 4.50 × B × C – 1.50 × A^2^ + 21.25 × B^2^ – 7.75 × C^2^
	*R*^2^ 0.9015, adjusted *R*^2^ 0.7242, adequate precision 9.978
CSA9 WVAC (%)	+10.48 – 2.56 × A + 1.64 × B – 0.17 × C – 2.53 × A × B – 0.025 × A × C – 1.99 × B × C + 3.16 × A^2^ – 1.57 × B^2^ + 2.19 × C^2^
	*R*^2^ 0.9202, adjusted *R*^2^ 0.7765, adequate precision 7.715

a A: Si/Ca molar ratio B: aging temperature (°C) C: reaction time (min).

**Table 4:** ANOVA and Fit Statistics for Models for Calcium Silica Aerogels Produced at pH 7.0 (CSA7)

	sum of squares		mean square		*F* value		*p*-value prob > *F*	
responses	SA	WVAC	SA	WVAC	SA	WVAC	SA	WVAC
model	37,636.18	349.83	4181.80	38.87	16.55	9.64	0.0033	0.0112
A	4753.13	21.39	4753.13	21.39	18.81	5.31	0.0074	0.0695
B	760.50	5.90	760.50	5.90	3.01	1.46	0.1433	0.2804
C	20,100.13	116.21	20,100.13	116.21	79.55	28.83	0.0003	0.0030
AB	196.00	6.33	196.00	6.33	0.78	1.57	0.4188	0.2657
AC	5112.25	52.35	5112.25	52.35	20.23	12.99	0.0064	0.0155
BC	1444.00	0.053	1444.00	0.053	5.71	0.013	0.0623	0.9132
A^2^	980.01	0.18	980.01	0.18	3.88	0.045	0.1060	0.8404
B^2^	22.31	64.03	22.31	64.03	0.088	15.89	0.7783	0.0105
C^2^	3950.16	93.95	3950.16	93.95	15.63	23.31	0.0108	0.0048
residual	1263.42	20.15	252.68	4.03				
lack of fit	1196.75	19.18	398.92	6.39	11.97	13.11	0.0781	0.0717
pure error	66.67	0.97	33.33	0.49				
cor total	38,899.60	369.99						

**Table 5:** ANOVA and Fit Statistics for Models for Calcium Silica Aerogels Produced at pH 9.0 (CSA9)

	sum of squares		mean square		*F* value		*p*-value prob > *F*	
responses	SA	WVAC	SA	WVAC	SA	WVAC	SA	WVAC
model	4523.35	180.77	502.59	20.09	5.08	6.41	0.0440	0.0273
A	612.50	52.43	612.50	52.43	6.20	16.72	0.0552	0.0095
B	703.13	21.52	703.13	21.52	7.11	6.86	0.0445	0.0471
C	171.13	0.24	171.13	0.24	1.73	0.078	0.2454	0.7910
AB	756.25	25.60	756.25	25.60	7.65	8.17	0.0396	0.0355
AC	182.25	2.50	182.25	2.50	1.84	7.973	0.2326	0.9786
BC	81.00	15.76	81.00	15.76	0.82	5.03	0.4069	0.0750
A^2^	8.31	36.91	8.31	36.91	0.084	11.77	0.7835	0.0186
B^2^	1667.31	9.14	1667.31	9.14	16.87	2.91	0.0093	0.1485
C^2^	221.77	17.65	221.77	17.65	2.24	5.63	0.1944	0.0637
residual	494.25	15.68	98.85	3.14				
lack of fit	476.25	2.72	158.75	0.91	17.64	0.14	0.0541	0.9276
pure error	18.00	12.96	9.00	6.48				
cor total	5017.60	196.45						

A, B, and C terms in the equation represent the first-order (linear) coded values, while A^2^, B^2^, and C^2^ are the second-order (square) coded values of the independent variables, and lastly, the terms of AB, BC, and AC represent the interaction of these variables. When the four responses created for two different samples in Table 3 are examined, the linear effect of the Si/Ca molar ratio only affected the surface area positively, while the WVAC was affected negatively. Aging temperature and reaction time have a negative effect on both surface area and WVAC, except for the CSA9 WVAC sample.

Adequate precision measures the signal-to-noise ratio, and a ratio greater than 4 is desirable. The ratio for all samples is greater than 4 and indicates an adequate signal. It means that these models have the capacity of providing acceptable performance according to the prediction.^18^

Additional confirmation of the suitability and adequacy of the models presented is that the *R*^2^ and *R*^2^ adjusted values are sufficiently close to each other. The *R*^2^ and *R*_adj_^2^ values of CSA7 SA are 0.9675 and 0.9091, respectively, while those of CSA7 WVAC are 0.9455 and 0.8475, and the *R*^2^ and *R*_adj_^2^ values of CSA9 SA are 0.9015 and 0.7242, respectively, while those of CSA9 WVAC are 0.9202 and 0.7765. It seems that the values are in reasonable agreement with each other.

Model terms’ significance is identified by the prob > *F* values, lower than 0.05 are significant, while higher than 0.1 is accepted as not significant.^19^ According to Tables 4 and 5, the quadratic model is suggested and well fitted for both responses of the surface area and WVAC with low prob > *F* value (*p*_model CSA7 SA_ = 0.0033, *p*_model CSA7 WVAC_ = 0.0112, *p*_model CSA9 SA_ = 0.0440, and *p*_model CSA9 WVAC_ = 0.0273). Likewise, the significance of the coded factors can be summarized as follows: *p*-values of A, C, AC, and C^2^ are significant for the surface area of CSA7, while C, AC, B^2^, and C^2^ are significant for the WVAC of CSA7 and *p*-values of B, AB, and B^2^ are significant for the surface area of CSA9, while A, B, AB, and A^2^ are significant for the WVAC of CSA9 (Table 4).

**Figure 1:**
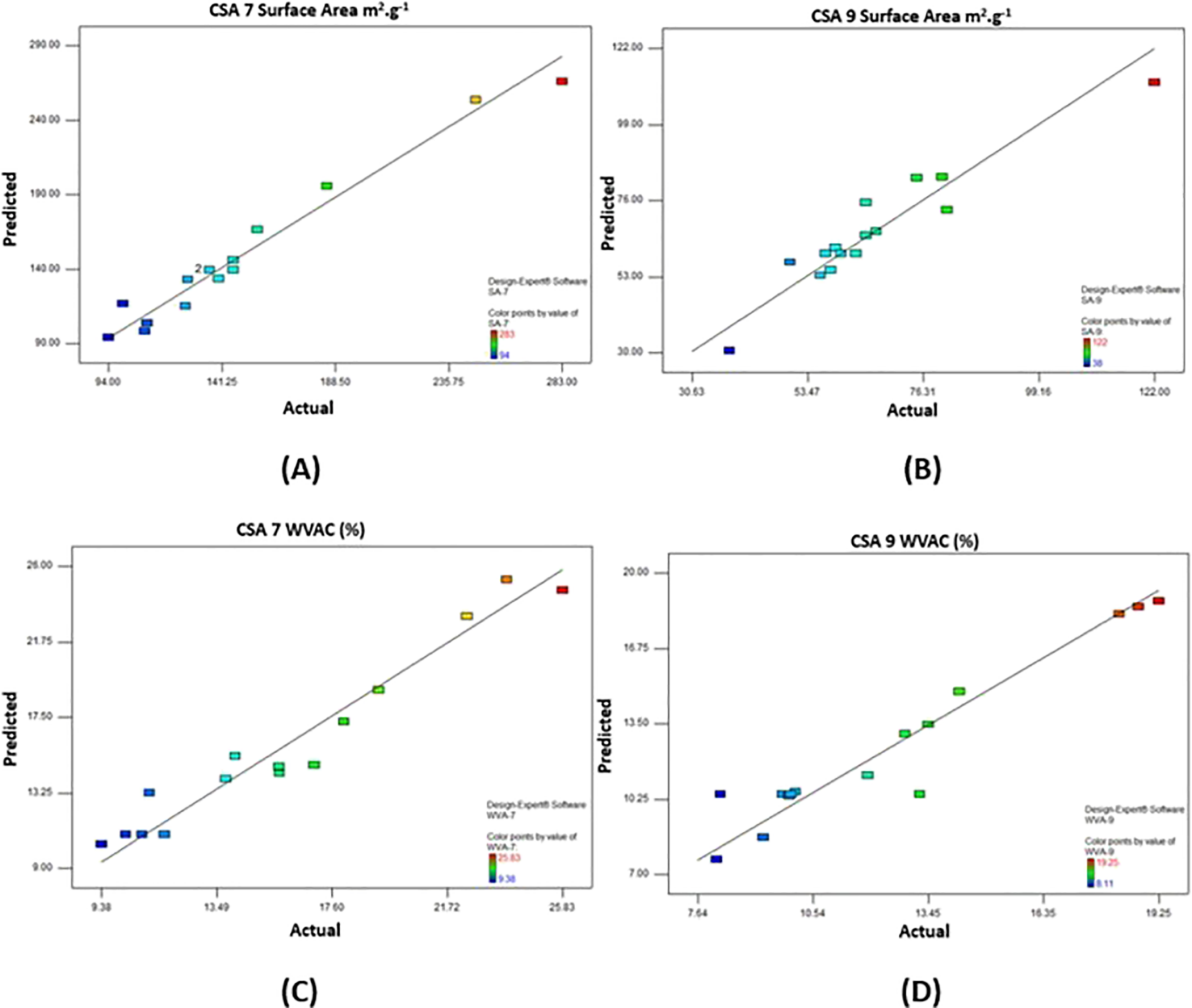
Actual vs predicted plots of CSA7 surface area (A), CSA9 surface area (B), CSA7 WVAC (C), and CSA9 WVAC (D).

Figure 1 shows the actual and predicted plots of all responses for CSA7 and CSA9. It can be understood from the figure that predicted values calculated from the equations given in Table 3 are in good alignment with the actual values because the data points are close to the straight line. This distribution around the line also proves the suitability of the proposed model.

### 3D Response Surface Plots

**Figure 2:**
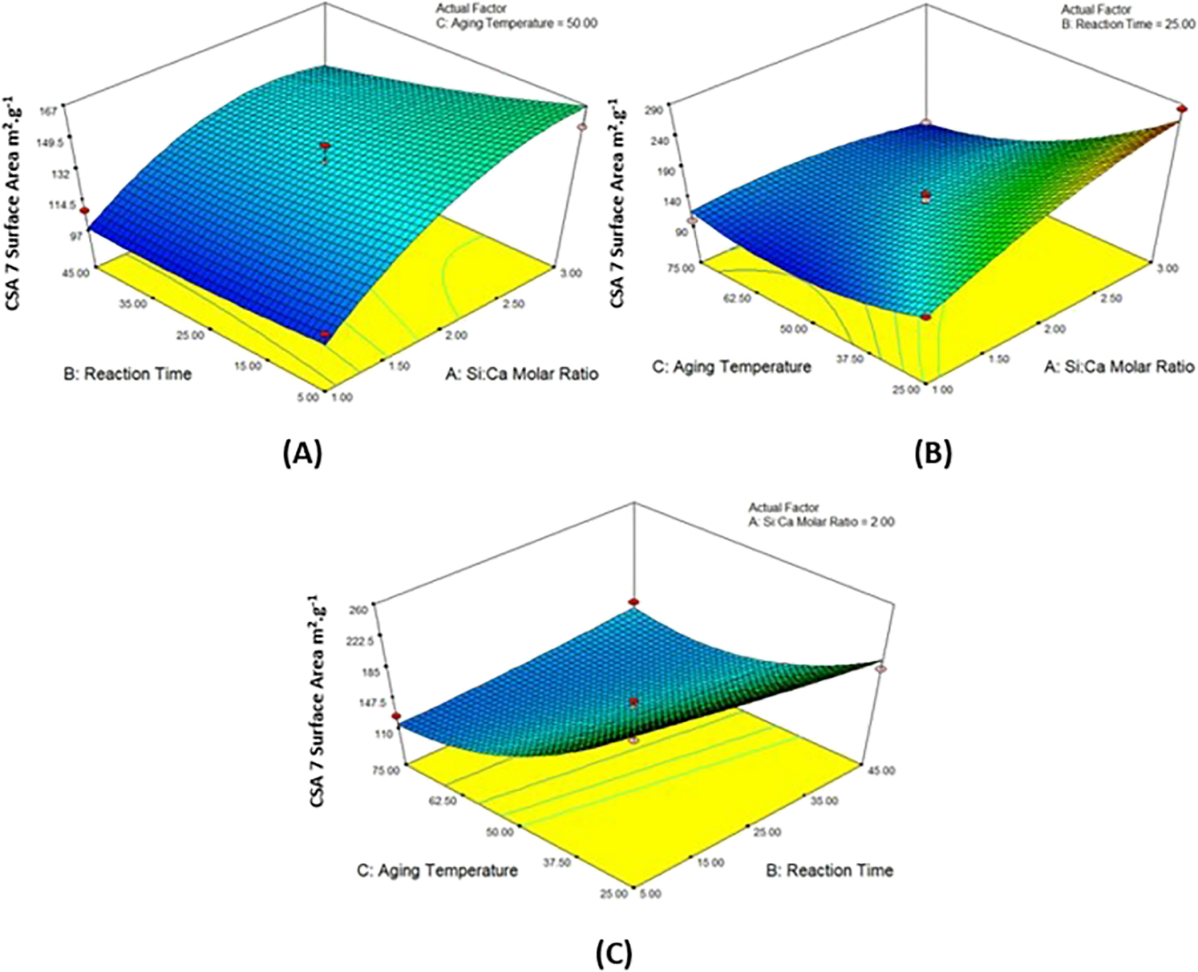
3D response surface plots of the (A) Si/Ca molar ratio and reaction time, (B) Si/Ca molar ratio and aging temperature, and (C) reaction time and aging temperature on the surface area of CSA7.

**Figure 3:**
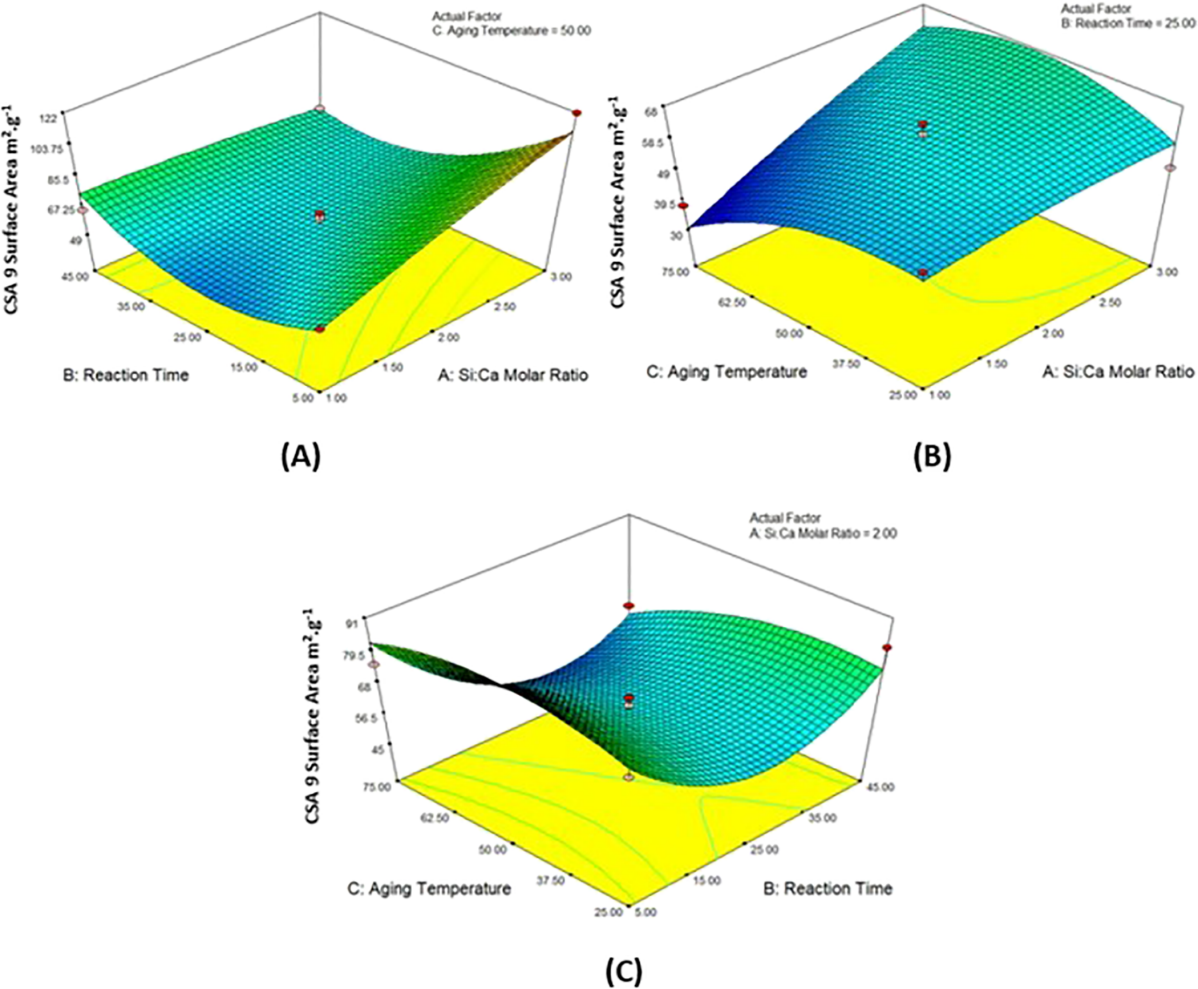
3D response surface plots of the (A) Si/Ca molar ratio and reaction time, (B) Si/Ca molar ratio and aging temperature, and (C) reaction time and aging temperature on the surface area of CSA 9.

**Figure 4:**
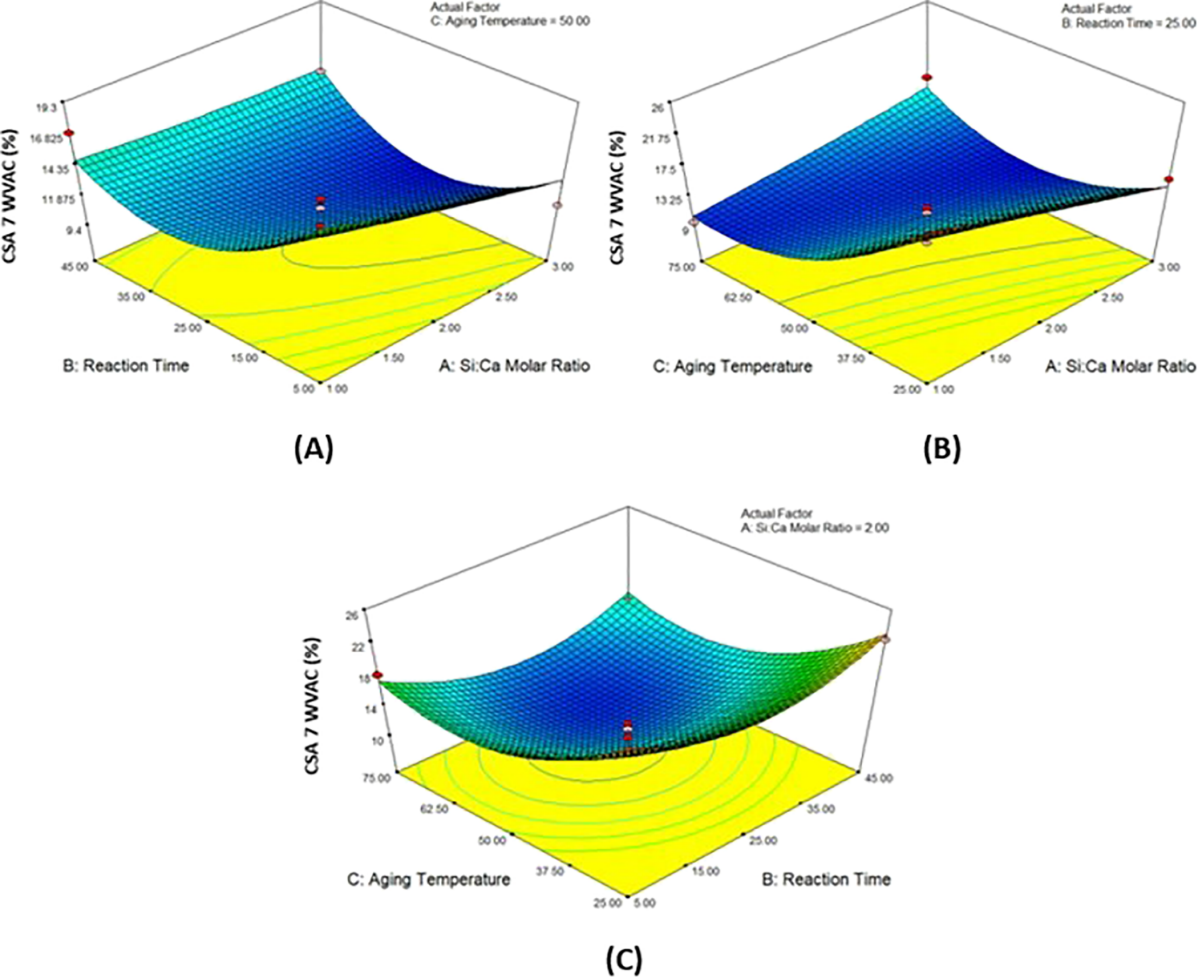
3D response surface plots of the (A) Si/Ca molar ratio and reaction time, (B) Si/Ca molar ratio and aging temperature, and (C) reaction time and aging temperature on the WVAC of CSA7.

**Figure 5:**
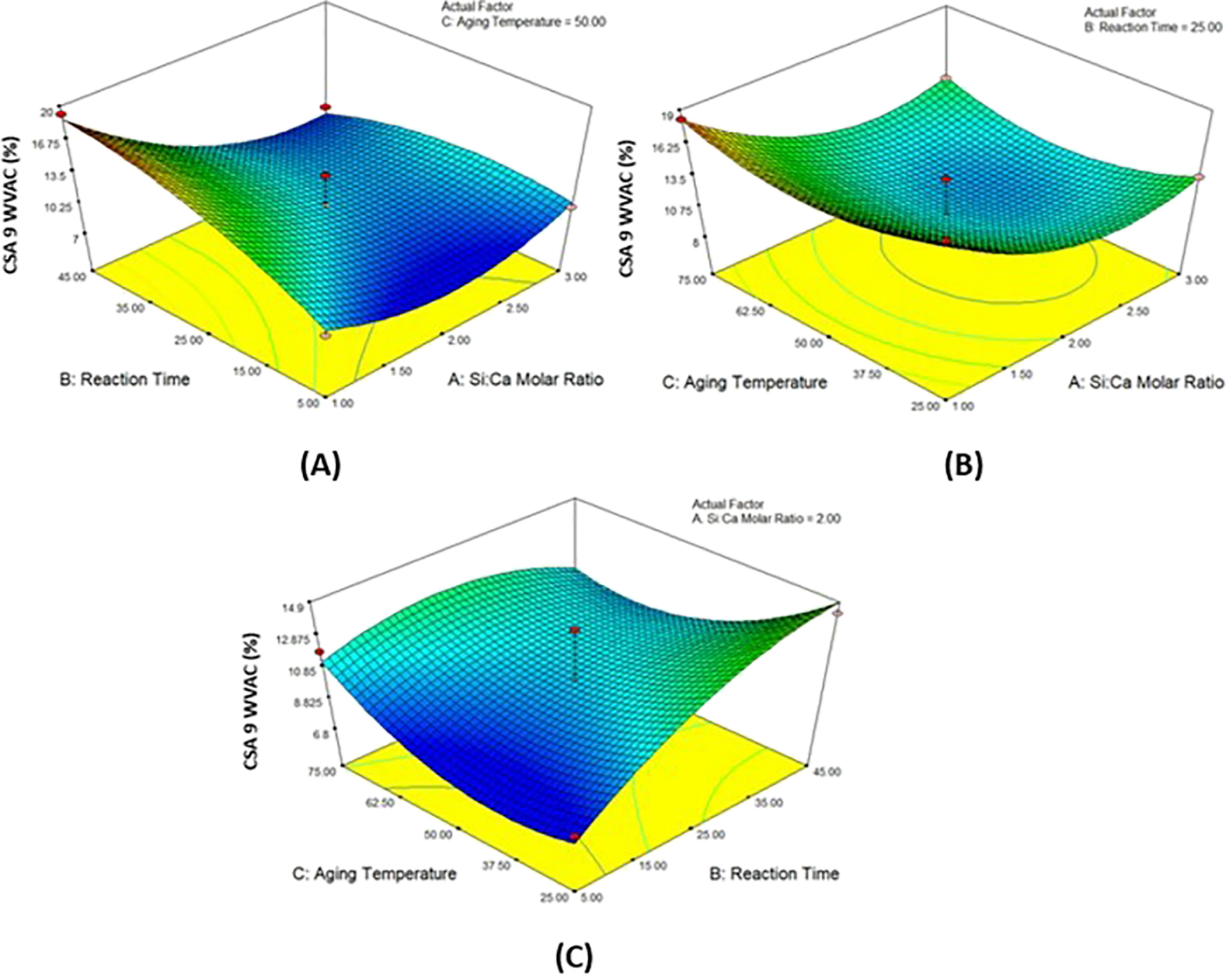
3D response surface plots of the (A) Si/Ca molar ratio and reaction time, (B) Si/Ca molar ratio and aging temperature, and (C) reaction time and aging temperature on the WVAC of the calcium silica aerogel produced with pH 9.0.

Figures 2–5 show that the mutual effect of two variables is examined, while the third variable is kept constant at the midpoint for each response.

The 3D response surface graphs of the parameters affecting the surface area of the calcium silica aerogel produced at pH 7.0 are examined in Figure 2. According to Figure 2A, at the constant aging temperature, the most effective parameter on the surface area is the molar ratio, and the changing reaction time does not have a positive or negative effect. In Figure 2B, it is seen that increasing the molar ratio had a positive effect on the surface area (from 94 to 283 m^2^/g) when the reaction time was kept constant, while the temperature change was not significant. In Figure 2C, it has been observed that there is a positive effect on the surface area at a constant molar ratio at low temperatures and low reaction time conditions (247 m^2^/g).

In Figure 3A, when the aging temperature was kept constant at the midpoint of 50 °C, the surface area reached the highest value at the point (122 m^2^/g) where the reaction time was the lowest (5 min), and the Si/Ca molar ratio was found to be the highest (3 mol). In Figure 3B, the surface area reaches its maximum value at the midpoint of the aging temperature (nearly 50 °C) with increasing value of the Si/Ca molar ratio, while the reaction time is kept constant (2 mol). Lastly, in Figure 3C, the reduction of the reaction time had a positive effect, while the aging temperature had no effect when the Si/Ca molar ratio was kept constant.

The response surface graphs of the WVAC of calcium silica aerogels produced at pH 7.0 are given in Figure 4. It was seen that the increase in reaction time and the temperature had a positive effect on water vapor adsorption (Figure 4A). In graph (B), it was noticed that keeping the Si/Ca molar ratio and temperature at low values resulted in an increase in the WVAC. In graph (C), it was seen that the reaction time and the change in the Si/Ca molar ratio did not have a significant effect.

Figure 5 shows the examination of the effect of the Si/Ca molar ratio, aging temperature, and reaction time on WVAC. It was found that in the condition of aging temperature kept constant, the Si/Ca molar ratio did not show a considerable effect, and the decrease in the reaction time caused an increase in the WVAC presented in Figure 5A. In Figure 5B, it is seen that increasing the Si/Ca molar ratio had a positive effect, and the aging temperature had no significant effect on WVAC when the reaction time was constant. In Figure 5C, when the Si/Ca molar ratio was kept constant, it was observed that the decreasing values of aging temperature and the increasing values of the reaction time had a positive effect on WVAC.

Since the aim of the study was to produce a calcium silica aerogel with high WVAC and high surface area, it was decided to continue with the calcium silica aerogels produced with pH 7.0, which has a higher surface area and high WVAC. It is understood that the results of both surface area and WVAC of calcium silica aerogels produced at pH 7.0 are more pleasing when the responses are evaluated collectively. The surface areas ranged from 94 to 247 m^2^/g for CSA7; these values varied from 38 to 122 m^2^/g for CSA9. The WVAC values for CSA7 ranged from 9.38 to 25.83%, while the WVAC values for CSA9 varied from 8.11 to 19.25%. Therefore, in the next part of the study, the detailed characterization of the optimized calcium silica aerogel produced according to the responses obtained in the RSM results and its behavior as an anticaking agent in the model food were examined.

**Figure 6:**
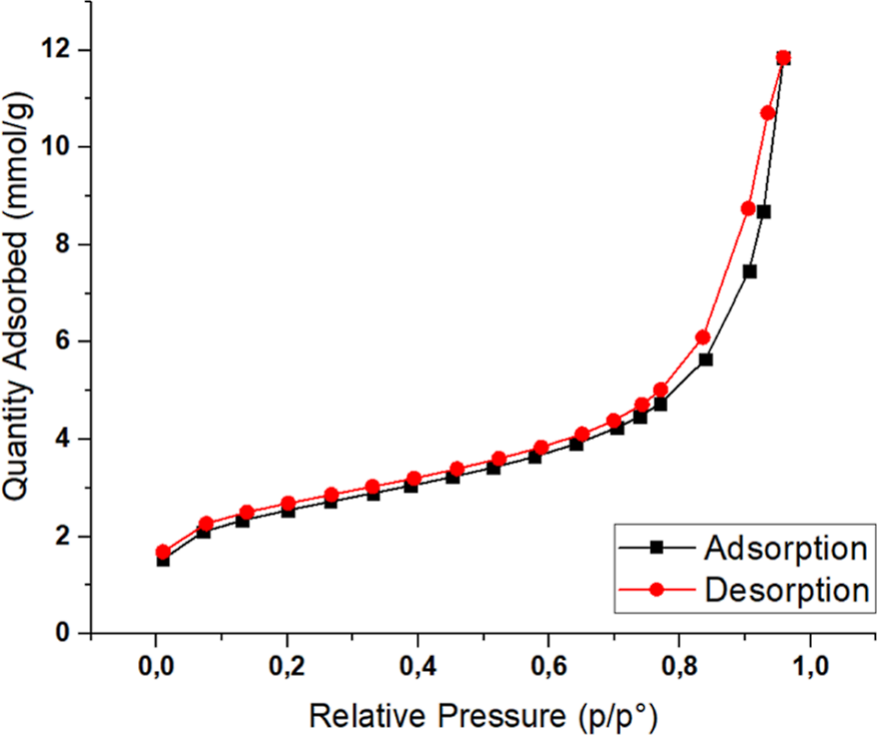
Nitrogen adsorption–desorption isotherm of CSA7.

### Nitrogen Gas Adsorption–Desorption Analysis

The specific surface area results of the calcium silica aerogels were the most important response of the study and were determined with nitrogen adsorption–desorption measurements at 77 K and calculated by BET analysis (Micromeritics Tristar II 3020, USA). Samples in the spherical tubes were settled in a degassing unit to purify the gases adhering to the sample surface; the degassing process occurred at 90 °C for an hour and right after 250 °C for 2 h in a nitrogen atmosphere. Besides that, pore volume and pore size were determined utilizing a different method named BJH in the same analysis device. N_2_ adsorption/desorption isotherms of the optimized CSA7 are given in Figure 6. These isotherms give information about the pore structure of the material. The state of the first increase indicates that adsorption is related to the presence of micropores in the mesh structure of the particles. According to the International Union of Pure and Applied Chemistry (IUPAC) classification, a type IV adsorption isotherm proves the presence of mesopores. The H1-type hysteresis loop demonstrates porous particles composed of agglomerates in the spherical form.^20^ The BET surface area of the optimized calcium silica aerogel was found to be 198 m^2^/g, the pore volume for BJH desorption was 0.203 cm^3^/g, and the pore size was 4.37 nm. The materials were classified by IUPAC according to the size of the pores they have, and materials with pores between 2 and 50 nm are named mesoporous.^21^ In this work, it was found that the optimized calcium silica aerogel has a mesoporous structure, and the result of 4.37 nm pore size proves a type IV adsorption isotherm.

**Figure 7:**
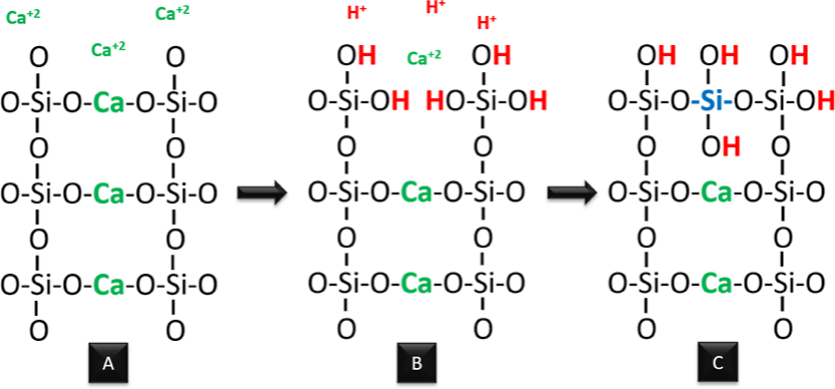
Addition of Ca^+2^ cations to the silica network (A), acidic solution addition to the medium and replacement of protons of H^+^ and Ca^+2^ cations (B), and calcium silica aerogel structure (C).

**Table 6:** Experimental Results of the Optimized Calcium Silica Aerogel Produced Using Optimum Process Parameters

sample name	Si/Ca molar ratio	aging temperature (°C)	reaction time (min)	surface area (m^2^/g)	WVAC (%)
optimized CSA7	2.42	25	5	198	17.56

Calcium silicate is described as a chain-silicate mineral. The covalently bonded silica network is interrupted and modified by Ca^2+^ cations that bonded to the structure weakly. Ca^2+^ is exchanged for hydrogen ions, and as a result of this, it is released into the solution and Si–OH formation occurred. Acid addition into the calcium silica aerogel reaction medium accelerated the formation of Si–OH while causing calcium depletion from the structure. The calcium ions that were depleted from the structure caused the formation of new pores and, as a result, there was an increase in the surface area (Figure 7). The surface area of calcium silicates is generally in a low range. In a study, the BET surface area of calcium silicate produced with wheat hull ash sodium silicate source was found to be 54 m^2^/g.^22^ Xue et al. prepared mesoporous calcium silicates for the controlled release of bovine serum albumin protein. The wet chemical method was used for the production of calcium silicates, and acid treatment was applied to the calcium silicate powder. A significant difference was detected between the surface area of the acid-treated and unmodified calcium silicates. The surface areas of the acid-treated calcium silicates were found to be 221, 333, and 356 m^2^/g for the pH values of 7.0, 4.5, and 0.5, respectively. The surface area was determined as 65 m^2^/g for unmodified calcium silicate. In addition, as a result of acid treatment at low pH values, SEM–EDS analysis showed that the presence of calcium gradually decreased and there was no calcium at pH 0.5.^23^ Although the preparation method, materials, and the studied pH ranges were different, the surface area results at pH 7.0 were found to be similar to this study. In this study, we reached the value of 198 m^2^/g for the surface area of the calcium silica aerogel produced at pH 7.0 and produced by ambient-pressure drying without using any solvent (Table 6).

### Inductively Coupled Plasma Atomic Emission Spectroscopy

Elemental analyses of the calcium silica aerogel were performed with ICP-OES. Prior to the analysis, calcium silica aerogel powders were added to an acidic solution and digested in a microwave.^24^ For this study, samples with a reaction time of 5 min were used since it was thought that the short reaction time in production had a positive effect on the surface area. Therefore, it is possible to examine the effect of aging temperature and Si/Ca molar ratio parameters on calcium content from the ICP-OES results. The main purpose of this analysis was to see the effect of the production pH value on the elemental contents of CSA7 and CSA9 produced using the same parameters (Table 7). In parallel with the results obtained in the study of Xue et al., it is possible to say that calcium binding decreases as the amount of acid used in production increases.^23^ CSA9 had the highest amount of calcium, but the surface area results and WVAC % values were not satisfactory. On the other hand, while calcium was present in sufficient amounts in CSA7, its surface area and WVAC values were also satisfactory.

**Table 7:** Content of Si, Ca, and Na in Calcium Silica Aerogels

	Si/Ca molar ratio	aging temperature (°C)	reaction time (min)	Si % wt	Ca % wt	Na % wt
CSA7	3	50	5	96.84	2.92	0.23
	1	50	5	92.23	7.22	0.56
	2	25	5	92.11	6.29	1.59
	2	75	5	94.83	4.55	0.63
optimized CSA7	2.42	25	5	86.88	11.37	1.75
CSA9	3	50	5	85.47	14.09	0.44
	1	50	5	82.57	17.29	0.14
	2	25	5	80.18	19.69	0.13
	2	75	5	82.27	17.58	0.15

### Fourier Transform Infrared Spectroscopy Analysis

**Figure 8:**
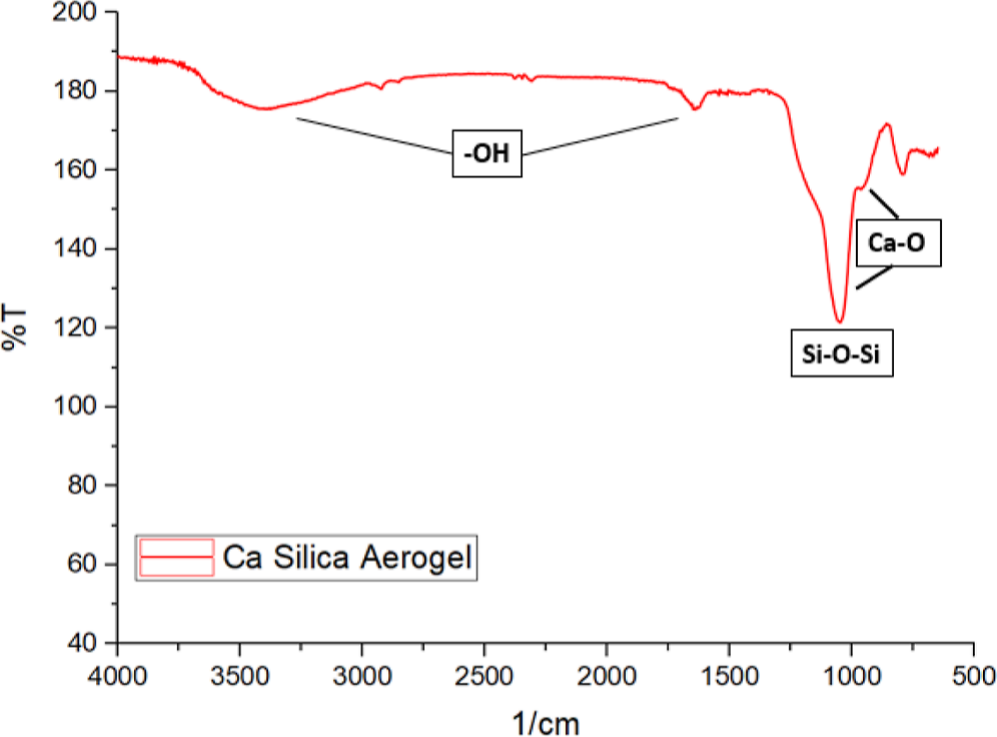
FTIR spectra of the optimized calcium silica aerogel.

The Fourier transform infrared spectroscopy with attenuated total reflection (FTIR–ATR) technique was used to investigate the chemical structure of the optimized calcium silica aerogel in the wavenumber range of 650–4000 cm^–1^. As shown in Figure 8, the main absorption bands of the calcium silica aerogel were detected at 840, 1000, 1100, 1600, and 3500 cm^–1^. Strong absorption at 1100 cm^–1^ was assigned to Si–O–Si asymmetric stretching vibrations.^25^ CaSiO_3_ was indicated at the wavenumbers 840 and 1000 cm^–1^. The band of 1000 cm^–1^ seemed to increase the intensity of the Si–O–Si band that was observed at 1100 cm^–1^.^26^ The absorption bands at around 1600 and 3500 cm^–1^ were related to −OH groups; they occurred due to the hydrophilic nature of the calcium silica aerogels, which means that the samples tend to capture the humidity from the air.^27,28^ Results show that calcium was successfully attached to the silica aerogel structure. Also, ICP-OES and SEM–EDS results can verify the existence of calcium in the structure.

### Scanning Electron Microscopy

**Figure 9:**
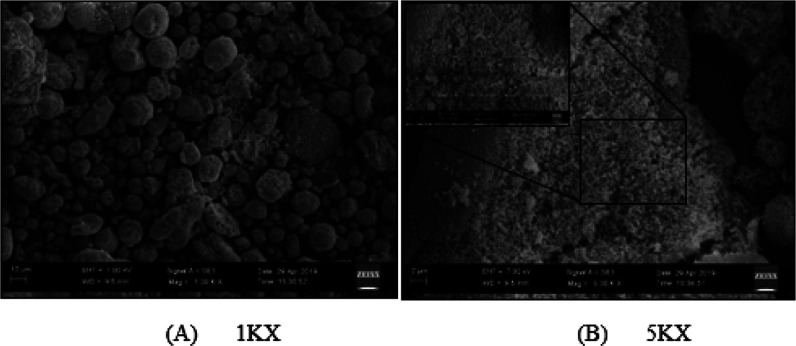
SEM images of optimized calcium silica aerogel particles (A: 1KX, B: 5KX, and 10KX magnifications).

The morphology of calcium silica aerogel particles, produced with the optimized parameters, can be seen in Figure 9. As can be seen in Figure 9A, calcium silica aerogel particles have different sizes of spherical shapes with some little surface irregularities. It can be noticed from Figure 9B that the particles have a porous structure. Every spherical large cluster in Figure 9A hosts small particles within it, and it can be witnessed very clearly in Figure 9B with the increase in magnification (1KX, 5KX, and 10KX).

### Particle Size Distribution of the Calcium Silica Aerogel

The particle size distribution of calcium silica aerogel powders was measured by using an LA-350 laser diffraction particle size distribution analyzer (Horiba, Japan). The measurement conditions were as follows: refractive index (RI): 1.46, imaginary (absorption): 0.010i, dispersant: water (RI = 1.333), circulation speed: 7, and ultrasound: 10 s.

**Figure 10:**
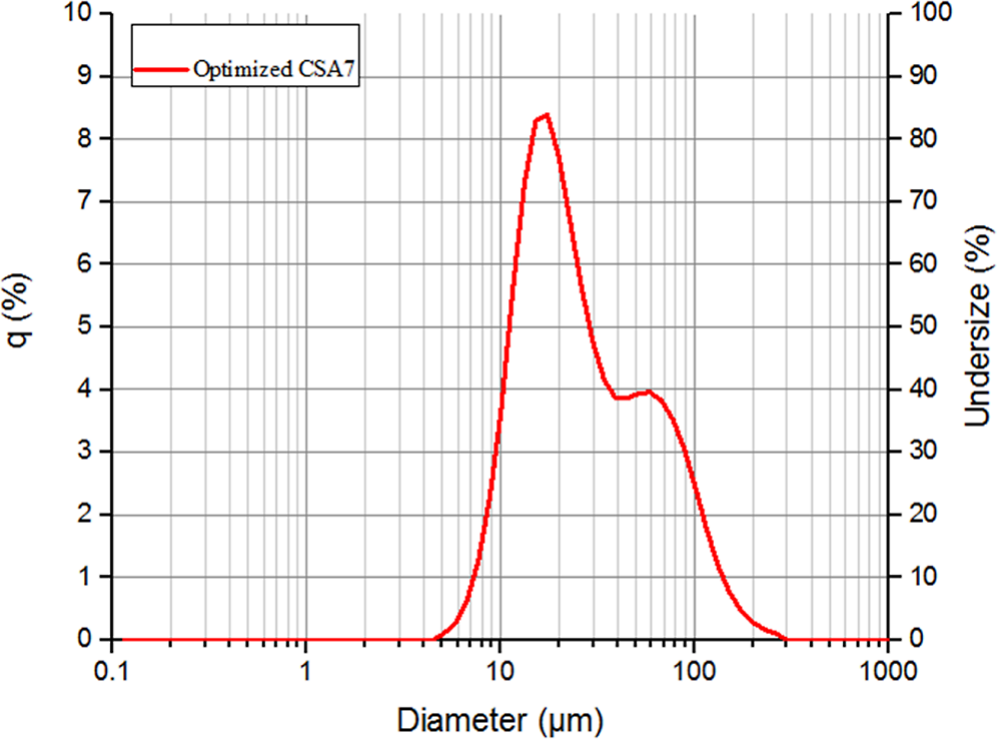
Particle size distribution of CSA7.

The cumulative diameter percentiles, d10, d50, and d90 (%), were determined as 10.51, 21.74, and 77.77 μm, respectively. Materials with a narrow particle size distribution showed better flow characteristics than materials with a wider particle size distribution. In powdered foods, the decrease in particle size increases the particle surface area and the formation of the sticky structure increases. In addition, an increase in moisture content also increases the strength of the adhesive structure due to the formation of liquid bridges and material flow between particles. As a result of the bridge between the particle and the container and the bonding force between the particles, a sticky structure emerges and the flowability decreases.^29^ It can be seen in Figure 10 that the optimized CSA7 produced in this study also has a very narrow particle size range. Thus, it shows promise that it can be used as a promising flow aid or anticaking agent.

### Density and Porosity Values of CSA7

**Figure 11:**
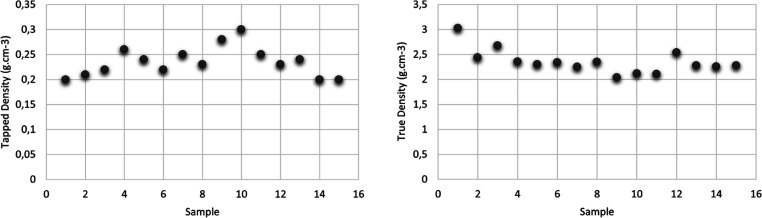
Tapped density and true density (particle density) values of CSA7.

Two different density values of CSA7 were determined. Figure 11 shows the tapped and true density values of 15 calcium silica aerogel powders produced at pH 7.0 in various parameters. The lowest density was generally achieved in products prepared at high aging temperatures. The highest density was reached at low aging temperatures. It was found that the reaction time and Si/Ca molar ratio have no significant effect on the tapped density. The tapped density values were in the range of 0.200–0.300 g cm^–3^. The true density values of all samples were very close to each other and in a range from 2 to 3 g cm^–3^. The presence of calcium ions bound to the samples can be considered as the reason for the difference in the true density values.

**Figure 12:**
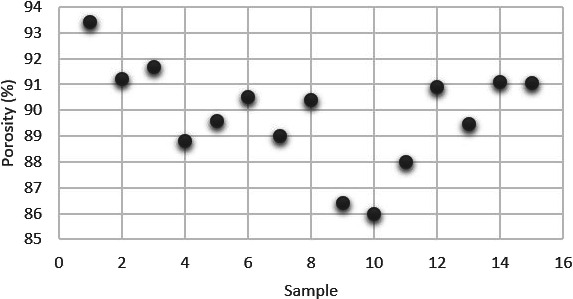
Porosity values of CSA7.

The 10-fold difference in the tapped density and particle density values indicates the highly porous structure of the particles. It was observed that the porosity values were in a wider range (from 85.99 to 93.42%) (Figure 12).

### Powder Flow Behavior/Test

**Figure 13:**
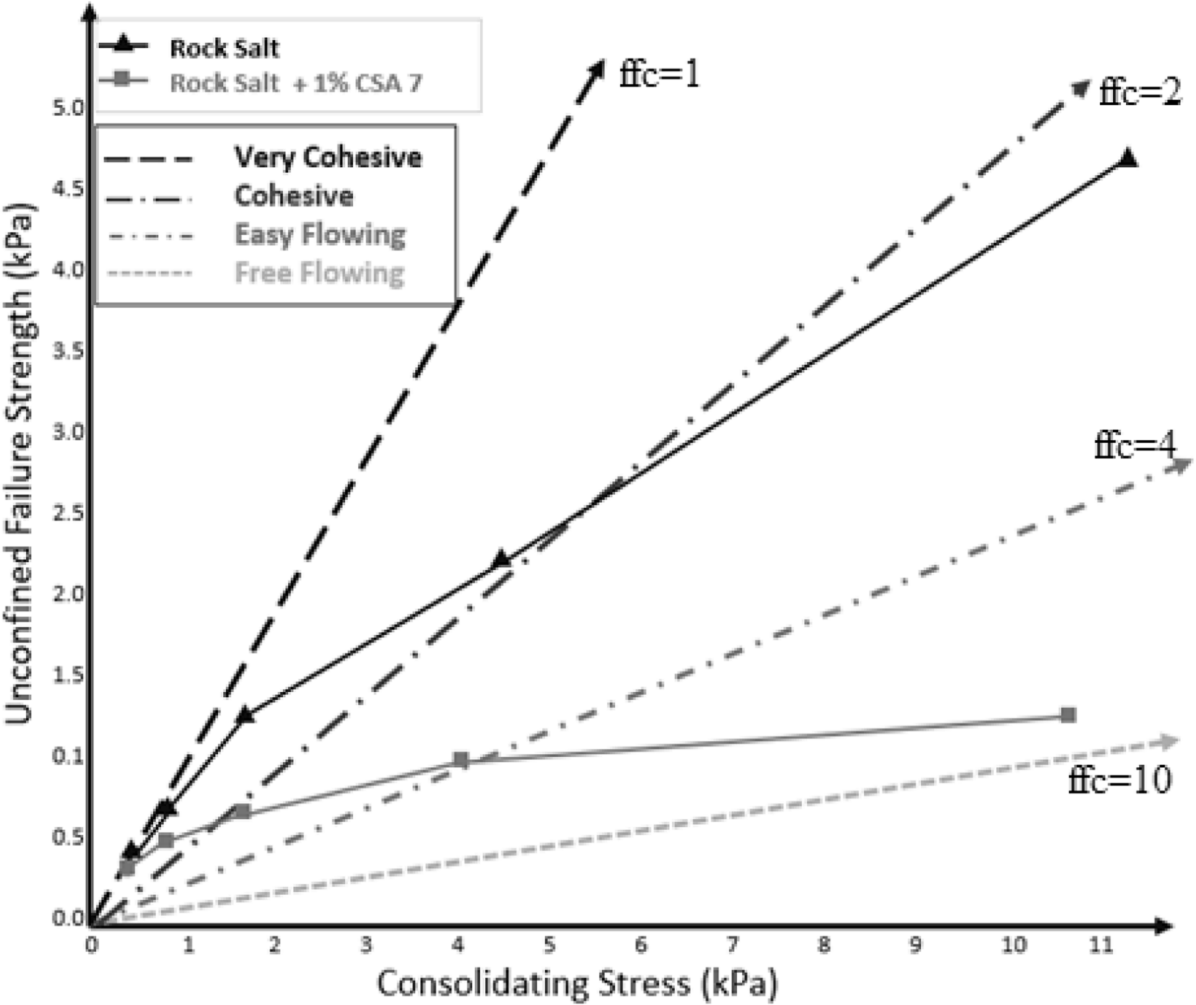
Flow function curves for CSA7 added rock salt.

Figure 13 shows the powder flow behavior of rock salt powder under consolidating stress with and without CSA7. The rock salt shows cohesive flow behavior under consolidation stress. The addition of optimized CSA7 powder at 1% (w/w, based on rock salt powder) improved the flow behavior from a cohesive region to an easy-flow region. As can be seen in Figure 13, the unconfined failure strength of the powder sample decreased under varying consolidation stress values after adding CSA7. This could be attributed to the adsorption of the moisture found in the environment and on the surface of the host particles of powder sample as well as sliding action caused by calcium silica aerogel particles in between the host particles which leads to the prevention of the formation of caking and, hence, improved flowability.

## Conclusions

Optimizing calcium silica aerogel production conditions by modeling is very valuable in terms of efficiency and quality. With the Box–Behnken design, the experimental design was easily prepared; thus, the effect of independent variables on calcium silica aerogel properties was quickly evaluated. It was found that the reaction time, Si/Ca molar ratio, and aging temperature significantly affect the surface area and WVAC of calcium silica aerogels. For CSA7, optimum conditions of the high surface area and high WVAC were obtained when the Si/Ca molar ratio was 2.42, the reaction time was 5 min, and the aging temperature was 25 °C. As a result, it has been shown that calcium silica aerogels with large surface area and high WVAC can be produced at pH 7.0 using low-cost sodium silicate under ambient-pressure drying conditions. Consequently, the data obtained from the powder flow test indicated that calcium silica aerogel powders have the promising potential to be used as an anticaking agent in powdered foods.
